# Antibiotic resistance risk assessment in Mymensingh, Bangladesh: Current scenario from human-animal-environmental interfaces viewpoint

**DOI:** 10.1016/j.heliyon.2024.e35878

**Published:** 2024-08-06

**Authors:** Zakaria Al Noman, Tasnia Tabassum Anika, Ummay Humaira Safa, Safaet Alam, Subarna Sandhani Dey, Md. Nurul Huda Bhuiyan, Mahbubul Pratik Siddique, Md. Mahmudul Hasan sikder

**Affiliations:** a Fruits and Food Processing and Preservation Research Division, Bangladesh Council of Scientific and Industrial Research, Rajshahi, 6206, Bangladesh; b Department of Pharmacology, Bangladesh Agricultural University, Mymensingh, 2202, Bangladesh; c Department of Pathology, Bangladesh Agricultural University, Mymensingh, 2202, Bangladesh; d Department of Media Studies and Journalism, University of Liberal Arts Bangladesh, Dhak, 1207, Bangladesh; e Chemical Research Division, BCSIR Dhaka Laboratories, Bangladesh Council of Scientific and Industrial Research (BCSIR), Dhaka, 1205, Bangladesh; f Department of Pharmaceutical Chemistry, Faculty of Pharmacy, University of Dhaka, Dhaka, 1000, Bangladesh; g Institute of Food Science and Technology, Bangladesh Council of Scientific and Industrial Research, Dhaka, 1205, Bangladesh; h Department of Microbiology and Hygiene, Bangladesh Agricultural University, Mymensingh, 2202, Bangladesh; i Food and Agriculture Organization, Bangladesh

**Keywords:** Prescription pattern, Human and veterinary drug, Antibiotic resistance, Antibiotic stewardship, One health approach

## Abstract

The imprudent use of antibiotics increases the environmental microflora's resistance to various drugs, particularly antibiotics. Prescription data is crucial for understanding antibiotic usage frequency and dosage. This health-focused study aims to analyze antibiotic prescription patterns in human and veterinary practices to identify emerging trends in environmental antibiotic resistance.

For this survey, A total of 6550 prescriptions were randomly collected from hospitals and pharmacies in Mymensingh sadar upazila, Bangladesh, between August and October 2022. Of these, 5123 (78 %) were for human cases and 1427 (22 %) for veterinary purposes. Photos of the prescriptions were taken and analyzed to understand prescribing habits. Additionally, 30 water samples from rivers, ponds, sewage, and households in Mymensingh City Corporation were collected to assess environmental antibiotic levels and resistance patterns of microorganisms.

The analysis showed that Cephalosporins were the most prescribed antibiotics, found in 570 (56.27 %) of human prescriptions and 230 (42.99 %) of veterinary prescriptions. Aminoglycosides had the lowest frequency, with 13 (1.2 %) for humans and 46 (8.6 %) for animals. Macrolides (12.24 %), carboxylic acids (1.87 %), and rifamycins (1.28 %) were only found in human prescriptions, while sulfa drugs (10.84 %), tetracyclines (5.42 %), and combinations of antibiotics (14.77 %) were only in animal prescriptions. Quinolones were prescribed 4.06 times more for humans, while aminoglycosides were used 3.54 times more for animals. Environmental samples showed *E. coli* had the highest resistance (MAR Value: 0.625) against eight antibiotics.

This study illuminates the human-animal prescription patterns that are influenced by environmental factors which drive antibiotic stewardship in Bangladesh. It is imperative for practitioners to exercise caution and adhere to guidelines when prescribing antibiotics, both in human and veterinary practices, given the alarming trend of antibiotic resistance. Additionally, measures must be taken to restrict the influx of antibiotics residue into the environment.

## 1 Introduction

In recent decades, antimicrobial resistance (AMR) has increased dramatically and has become a major global public health issue [1,2] and World Health Organization (WHO) identified AMR as the main global health challenge to human, animal, and environment [3]. The demand for antibiotics is on the rise in both low- and middle-income countries as well as developed nations who have adapted to globalization. Globally, antibiotic use has increased by 46 %, but in low and middle-income countries, it has skyrocketed by 76 % over the past two decades [4]. This increase reflects a variety of classifications of antibiotic usage patterns, including rational or irrational uses (abuse, misuse and abuse), which contribute to antimicrobial resistance (AMR) [5]. The geo-climatic changing pattern may influence the occurrence of zoonotic and emerging diseases which may also initiate the necessity of antibiotic consumption [6,7].

AMR is rising rapidly due to uneven policies of antibiotic use, antibiotic void, palliative diagnosis and treatment process, conventional preventive care and management as well as many factors [[8], [9], [10]]. AMR spreading rate is higher in developing countries due to irrational antibiotic prescribing patterns, poor sanitation, endemic infections, malnutrition, lack of standard guidelines and awareness, ease of accessibility on antibiotics etc. [[11], [12], [13]] but sufficient information on this issue is limited [14]. In fact, the emergence and spread of AMR is mainly from environmental spillage of antibiotics from various sources like human and veterinary medicals, crops, and aquaculture farming [15,16] Therefore, AMR is one of the major issues of ‘One health’ which could be a future pandemic due to connectivity and complexity among different trends of life [17]. One Health is an integrated, unifying approach that aims to sustainably balance and optimize the health of people, animals, and ecosystems. It recognizes the health of humans, domestic and wild animals, plants, and the wider environment (including ecosystems) are closely linked and interdependent [18]^.^

The AMR is an emerging issue in Bangladesh [19] due to geo-climatic influence on disease epidemiology, weak health management system, multiple pathways of antibiotic consumption in human-animal-agricultural practices [[20], [21], [22]]. Bangladesh has recently approved a national strategic plan on antibiotic prescribing challenges to avoid future pandemic [23]. The environment is one of the neglected but prominent portion for AMR study. Environmental contamination contributes most efficiently in the selection of resistant bacterial populations and in antimicrobial resistance genes (ARGs) exchange through mobile genetics elements [1,24]. There is also alternative factors which may facilitate the antimicrobial resistance phenomenon [[25], [26], [27]].

Antimicrobial resistance is a multi-facet problem and need multi-pronged approach to combat this situation [28]. Moreover, the ‘One Health’ concept is still theoretical in developing countries, like Bangladesh [29,30]. Therefore, the AMR should be addressed at human-animal-environment interface for the antibiotic stewardship in developing countries and this snapshot study was aimed to draw a real scenario of antibiotic prescribing pattern in between human and veterinary disease management and to assess environmental concern for understanding the antimicrobial resistance stewardship. The antibiotic prescribing pattern and irrational use of antibiotics in both human and veterinary clinical cases have been contributing to the development of pool of resistant isolates and residues in the biotic and abiotic components of the environment [31].The antibiotic resistant microorganisms, residual effect and resistance genetic materials have been considering as the environmental pollutants which is a serious public health crisis [32]. Hence, the proper recording of antibiotic use, prescribing patterns analysis and environmental resistant bacterial isolates detection and characterization could be helpful for the policy makers to develop a sustainable framework for combating the antibiotic resistance threat at national and global level [33].

Antibiotics have been restricted, even, antibiotics of several classes have been banned in many countries, however, in developing countries, antibiotics have still been prescribing to control infectious diseases or as growth promoter [34]. According to the previous study, antibiotic overprescribing as the major constraint for antibiotic stewardship intervention, and found that 56 % prescription for human cases contained one or two antibiotics in Addis Ababa, Ethiopia [35]. In Bangladesh, it was reported that the clinician did not follow the standard guideline of antibiotic prescribing [36]. Moreover, a point prevalence survey on 1417 enrolled patients and 78 % of patients received at least one antibiotic during the survey period [37]. Again, the antibiotic prescribing patterns was analyzed to investigate the influences of developing AMR pathogen over time [38]. To obtain the specific objective, antibiotic prescribing pattern in human and veterinary practices with probable associations and potential roles of antibiotic on environment for antibiotic stewardship is thoroughly investigated in this study. The environment increases complexity by triggering many drivers, including pathogenic adaptability to unfavorable circumstances, genetic variables associated with antimicrobial resistance, and antimicrobial residue. However, the environment has not been taken into consideration in antimicrobial resistance studies to date [4,5,39]. Among the environmental components like water, soil, plants and so on are interlinked with each activity of our life. Using of antibiotic in one point may impacts on the whole for initiating the antimicrobial stewardship [5,10]. Veterinary antibiotics are using on livestock as growth promoter, preventive measure or disease management which may contaminate the environment by direct ways such as manure in a form of antibiotic residual surplus and metabolites and indirect ways such as AMR properties may transfer to human through food chain which also contaminate the environment by following the same process [40]. The longitudinal and horizontal interchange of antibiotic resistance genetic materials of micro-organisms from multiple environmental constituents also initiates the potential ecological risk of antibiotics [41].

So, in this work, a spotlight has been drawn on human-animal prescription patterns influenced by environmental attributes resulting in antibiotic stewardship in Bangladesh to ameliorate the alarming trend of antibiotic resistance. The finding of this study would help drug regulatory body and concerning authorities, healthcare experts (in both human and veterinary), policy makers to develop sustainable strategies for strengthening the ‘One Health’ policy in Bangladesh.

## 2 Methodology

### 2.1 Study design

**Fig. 1:**
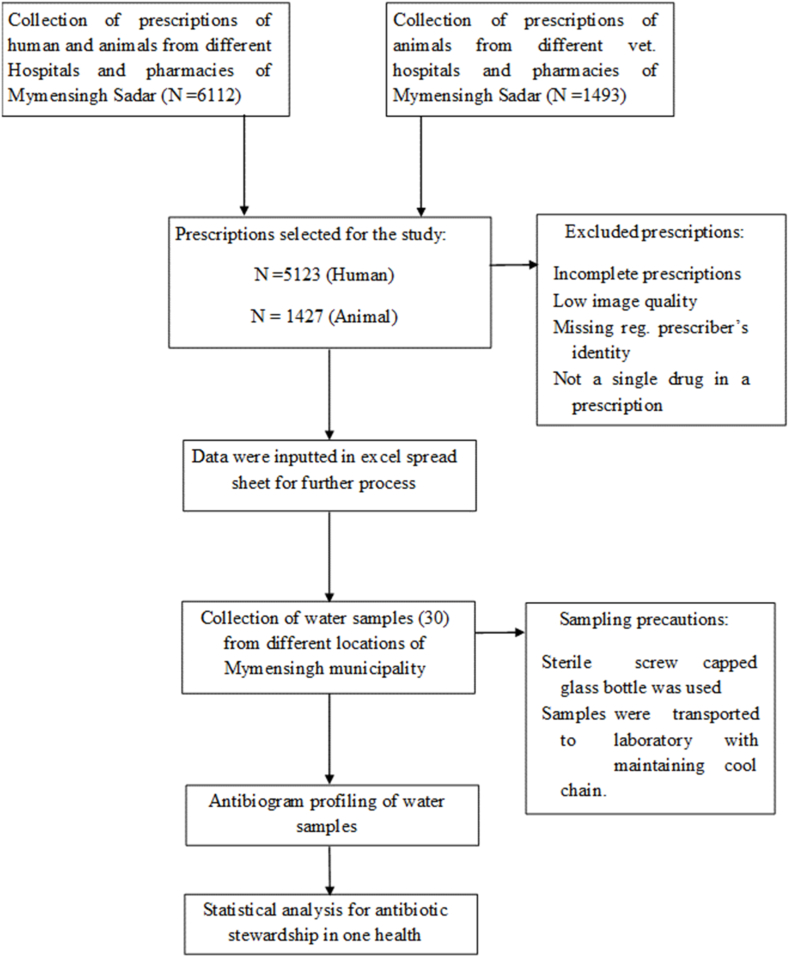
Sketching of the study methodology.

This study was a snapshot investigation of the prescribed antibiotics in Mymensingh district, Bangladesh (as shown in Fig. 1), conducted according to an approved protocol by Bangladesh Agricultural University, Mymensingh-2202. As computer-based prescriptions are not yet universally practiced throughout Bangladesh, we captured photographs of paper prescriptions from registered human physicians and veterinarians only (including those working within both government services and private practices) using smartphone cameras at various human and veterinary hospitals as well as pharmacies located in sadar upazila, Mymensingh. The collected prescriptions underwent thorough examination by expert pharmacologists to ensure their validity. For this study, only completed prescriptions containing all essential components with clear information were selected for analysis; incomplete or low-quality images lacking sufficient information were discarded. Each antibiotic present or absent in each prescription was scored using a counting scale for descriptive data analysis purposes.

**Fig. 2:**
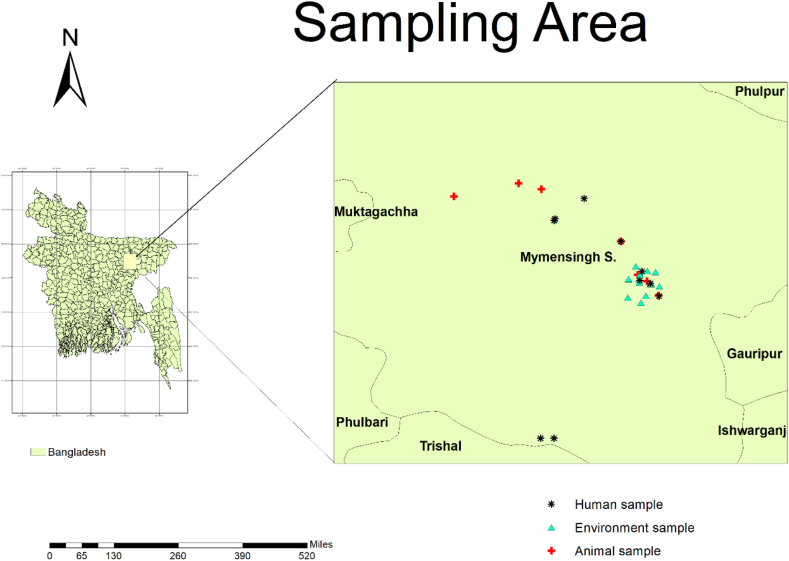
Sampling area for understanding the antibiotic stewardship in Mymensingh district, Bangladesh.

The sample size was established using the Raosoft software-based statistical tool [34] with a 95 % confidence interval and 5 % margin of error, resulting in an estimated minimum sample size of 377. Between August and October of the year 2022, we collected and analyzed a total of 5123 human prescriptions from two government hospitals, four private hospitals, and thirteen pharmacies as well as 1427 veterinary prescriptions from two government veterinary hospitals, two private veterinary hospitals, seven pharmacies, and thirty-five farms located within Mymensingh's Sadar upazila. The convenient prescriptions were collected after having written consent by the concerned patients or farm owners. Additionally, we randomly gathered water samples from various sources such as rivers, ponds, sewage systems, households etc., throughout the Mymensingh City Corporation territory between mid-October to November in the same year. For this study specifically we considered thirty water samples as per permission we could manage but tried to cover the biggest portion of study area. The sampling sites' geographic locations were plotted on Fig. 2 for visual aid purposes.

### 2.2 Environmental sample processing and enrichment

The water samples from each sampling point were collected in sterile glass bottle. Bottles were cleaned with detergent (absent of anti-bacterial agents) and autoclaved in 121 °C for 15 min. About 100 mL of water was collected in the bottles from each sampling site, leaving enough air in the bottle for agitation and mixing. Water samples were filtered through 0.2 μm nitrocellulose membrane filters. The filter papers were placed in a 90 mL sterile nutrient broth and incubated at 37 °C for overnight enrichment. Processing of samples was started soon after collection (typically within 12–16 h of collection).

### 2.3 Isolation of bacterial strain by selective culture method

**Fig. 3:**
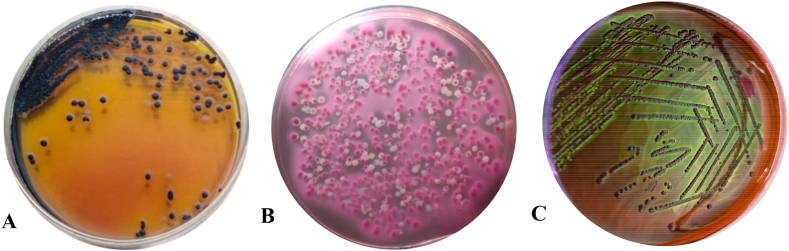
**A.***Salmonella* sp. was denoted as Black centered colonies on Xylose lysine deoxycholate (XLD) agar plate; **B.** pink colonies of *E. coli* at MacConkey agar plate; **C.** Greenish metallic sheen of *E. coli* at Eosine methylene blue (EMB) agar medium.

0.1 mL of overnight enriched samples were then inoculated by spread plate method on Xylose lysine deoxycholate (XLD), MacConkey and Mannitol salt agar media for detection of *Salmonella* sp., *E. coli* and *Staphylococcus aureus* respectively and incubated at 37 °C temperature for 24 h **[**Fig. 3(A–B)**].** For further confirmation of *E. coli*, pink colonies from MacConkey agar were inoculated on Eosine methylene blue (EMB) agar medium **[**Fig. 3**C].**

### 2.4 Purification and preservation of bacterial strain

Standard selective agar plates containing 20–200 colonies were selected and bacterial colonies with selective characteristics were further streaked for purification and some selected colonies were further streaked on nutrient agar medium for preservation.

### 2.5 Antibiotic susceptibility assay

Antibiotics were used to characterize bacterial strains based on their sensitivity and resistance toward the different groups of antibiotics. Antibiotic disks with a specified concentration of a particular antibiotic dose were used to obtain the bacterial strain's sensitivity or resistance towards the specific antibiotic. The antibiotic sensitivity performance of isolates from water were checked through Kirby-Bauer Disk Diffusion Susceptibility Test Protocol and the zone of inhibition was measured in mm [42]. Tetracycline 30 μg (T), Ampicillin 10 μg (AM), Ciprofloxacin 5 μg (CIP), Ceftriaxone 30 μg (CTR), Trimethoprim 25 μg (COT), Chloramphenicol 30 μg (C), Gentamicin 10 μg (GEN) and Erythromycin15 μg (E) were checked in this study.

### 2.6 Inoculum preparation

According to the standard guideline described by the Clinical and Laboratory Standards Institute (CLSI, 2020), 0.5 McFarland turbidity standard was prepared. The preserved isolates were inoculated on nutrient agar plates and incubated overnight at 37 °C. At least 2–3 well-isolated colonies were selected from the Nutrient Agar plate and transferred into Mueller-Hinton Broth (MHB) using a sterile loop. Each tube of MHB containing 5 mL media were incubated at 37 °C on the shaker for 4–5 h after inoculation.

The broth cultures were incubated at 37 °C to achieve the 0.5 McFarland standard. The turbidity of the actively growing broth culture was adjusted with sterile broth to obtain turbidity optically comparable to the point of the 0.5 McFarland standards. *Escherichia coli* 25922 was used as a control.

### 2.7 Inoculation of test plates

Within 15 min of adjusting the turbidity of the test culture, a sterile cotton swab was dipped into the adjusted suspension. The swab was rotated several times and pressed firmly on the inside wall of the respected culture tube above the culture to remove the excess culture from the swab. The dried surface of a Mueller-Hinton agar plate was inoculated by streaking the swab over the entire sterile agar surface. This procedure was repeated by streaking two more times rotating the plate at 90° angles each time to ensure an even distribution of inoculums. As a final step the rim of the agar was swabbed. The procedure was done under laminar air flow to avoid contamination.

The lid was left ajar for 3–5 min but no more than 15 min, to allow for any access surface moisture to be absorbed before applying the drug impregnated disks.

### 2.8 Reading plates and results interpretation

After 16–18 h of incubation, each plate was examined for the zone of inhibition, circular with a confluent lawn of growth. The diameters of the zones of complete inhibition (judged by the unaided eye) were measured, including the diameter of the disc. Zones were measured to the nearest whole millimeter. The faint growth of tiny colonies, which can be detected only with a magnifying lens at the edge of the zone of inhibited growth, was ignored. The disk diffusion test was replicated thrice for understanding the antibiotic sensitivity in comparison with the CLSI guideline [43].

### 2.9 Data analysis

The study area mapping was prepared with ArcGIS software (10.8 version). For analyzing prescriptions, the data input was made into a spreadsheet (Microsoft Excel, 2019 version) and descriptive analysis was performed using statistical software (SPSS, 2019).

### 2.10 Ethical considerations

The research protocol has been approved by Animal Welfare, Experiment and Ethics Committee, Bangladesh Agricultural University, Mymensingh-2202 (AWEEC/BAU/2021(63), date: 23.12.2021). Written ethical consent was taken before capturing the prescription from respondents and animal owners. Few ethical consents were provided in supplementary section as examples. We also ensured the confidentiality of identity for all the respondents, animal owners, hospitals and pharmacies related to this study. We were concerned on environment samples for proper authorities’ consideration.

## 3 Results

### 3.1 Antibiotic prescribing pattern

Among the prescribing pattern of antibiotics, human 1013 (19.77 %) and veterinary 535 (37.49 %) prescriptions contain at least an antibiotic. In human prescription, 81.04 % antibiotics were prescribed for adults and rest or children (18.95 %). Large animal group (Bovine) was the largest consumer (58.50 %) followed by caprine (24.67 %), avian (11.58 %) and pet animal (5.23 %). Antibiotic administration pattern reveals that human practitioners prefer oral route (80.35 %) but parenteral route (96.07 %) was more practiced in animals along with β-lactam inhibitor (11.02 %). The descriptive results of prescription were represented into Table 1.

**Table 1:** Descriptive analysis of antibiotic prescribing pattern.

Prescription type	Prescription containing Antibiotic	Percentage (%)	Antibiotic consuming category	Number of consumers	Percentage (%)
Human (n = 5123)	1013	19.77	Adult	821	81.05
			children	192	18.95
Veterinary (n = 1427)	535	37.49	Bovine	313	58.50
			Caprine	132	24.67
			Avian	62	11.59
			Pet animals	28	5.24

**Fig. 4:**
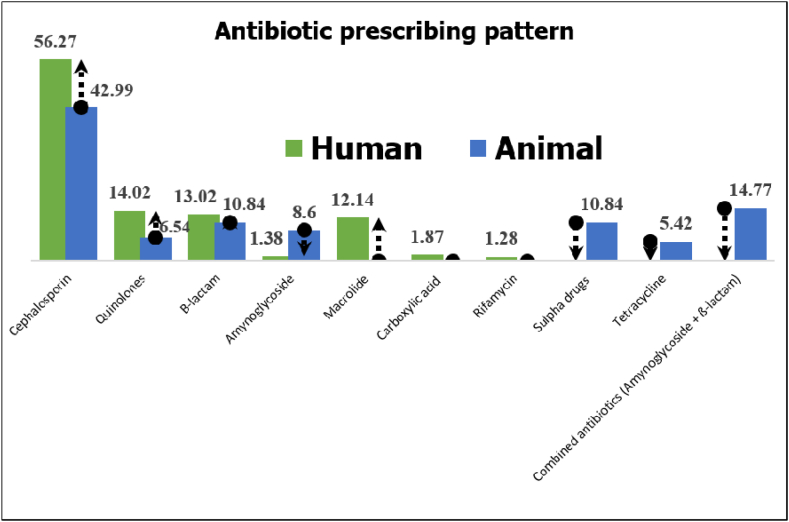
Comparative behavior of antibiotic Consumption in between men and animal.

The antibiotic comparative prescribing pattern in between human and animals were also analyzed (Fig. 4). Cephalosporin was prescribed as the most common in human 570 (56.27 %) and 230 (42.99 %) in veterinary prescriptions. Quinolones 142 (14.02 %) and 35 (6.56 %), β-lactams 132 (13.02 %) and 58 (10.84 %) as well as aminoglycoside 13 (1.2 %) and 46 (8.6 %) were common antibiotics for both practices. Macrolide 124 (12.24 %), carboxylic acid 19 (1.87 %) and rifamycin 13 (1.28 %) were found only in human, Whereas sulpha drugs 58 (10.84 %), tetracycline 29 (5.42 %) as well as combination of two group of antibiotics (14.77 %) for animal.

Among the common antibiotics prescribing rate analysis in between human and animals, quinolones ranked top with 4.06 times higher in humans in relation to animals followed by cephalosporins (2.48) and β-lactams (2.27) respectively where aminoglycosides were prescribed 3.54 times higher in animals in compared to human.

### 3.2 Prevalence of bacterial strains

The occurrence of *E. coli*, Salmonella sp., and Staphylococcus sp. registered a rate of 100 %, 86.66 %, and 73.33 % respectively in the diverse environmental (water) samples tested (Table 2). The bacterial strains were verified by means of specific cultural characteristics analysis through particular culture methodologies.

**Table 2:** Prevalence of bacterial isolates from different sampling sites.

Source of Environmental Sample	Total environmental (water) sample	*E. coli*	*Salmonella* sp.	*Staphylococcus* sp.
Sewage sample	17	17	14	13
Household sample (home, restaurant and etc.)	8	8	8	7
Fresh water sample (pond, river and etc.)	5	5	4	2
Total	30	30 (100 %)	26 (86.66 %)	22(73.33 %)

### 3.3 Antibiotic susceptibility test

In this study, the antibiogram profile was analyzed utilizing nine distinct antibiotics from eight unique groups. The disc diffusion method on Muller Hinton agar media was employed to observe the pattern of antibiotic susceptibility. The result is summarized in Table 3.

**Table 3:** Antibiogram pattern of environmental bacterial isolates.

Isolate	Zone of inhibition (ZI) in mm								Total resistant antibiotic	MAR index
	T	AM	CIP	COT	CTR	C	GEN	E		
I_1_	14 ± 1 IR	0 ± 0 R	14 ± 1 R	6 ± 0 R	9 ± 0 R	20 ± 1 S	11 ± 0 R	15 ± 1 S	5	0.625
I_2_	16 ± 2 S	0 ± 0 R	25 ± 1 IR	18 ± 1 S	24 ± 2 IR	19 ± 1 S	15 ± 2 S	0 ± 0 R	2	0.25
I_3_	18 ± 2 IR	0 ± 0 R	12 ± 1 R	23 ± 0.5 S	21 ± 1.5 S	21 ± 1 S	18 ± 2 S	17 ± 0 IR	2	0.25

*I_1_ = *E. coli,* I_2_= *Salmonella* sp.*,* I_3_= *Staphylococcus* sp., S = sensitive, IR = intermediately resistant, R = resistant, MAR index = Multiple antibiotic resistant index, T = Tetracycline 30 μg, AM = Ampicillin 10 μg, CIP=Ciprofloxacin 5 μg, CTR=Ceftriaxone 30 μg, COT = Trimethoprim 25 μg, C=Chloramphenicol 30 μg, GEN=Gentamicin 10 μg, E = Erythromycin 15 μg.

In this research, *E. coli* exhibited the highest degree of resistance (against five out of eight) compared to other microorganisms. This highlights the crucial role played by the environment in addressing the impending threat of antimicrobial resistance, which is far more significant than human and animal health management alone. Unfortunately, due to an uneven sample size between prescription and environmental data sets, no statistical testing could be performed. Nevertheless, it was observed that 23.64 % of prescriptions showed positivity in both human and veterinary practices while a staggering 86.67 % of environmental isolates demonstrated resistance or intermediate resistance to two or more antibiotics at minimum.

## 4 Discussion

The objective of this study employing a one-health approach is to gain an understanding of the antibiotic prescription patterns in disease management, encompassing both human and veterinary practices, while considering its impact on the environment for effective antibiotic stewardship.

The antibiotic prescribing pattern in both human and veterinary practices was studied where cephalosporin was the most common prescribing antibiotics, 56.27 % and 42.99 % in human and veterinary practices followed by quinolones 14.02 % and 6.56 %, β-lactams 13.02 % and 10.84 % as well as aminoglycoside 1.2 % and 8.6 %. But macrolide, carboxylic acid and rifamycin were found only in human, whereas sulpha drugs, tetracycline for animal. The result revealed a red alarm to understanding the havoc of upcoming antibiotic-resistant pandemics across the country. It is high time to stop misuse, abuse and overuse of antibiotics in each and every arena around us for better public health concerns. Understanding the potential of antibiotics in the environment for the antibiotic stewardship was focusing point to achieve concluding remarks.

In our study, among the 19.77 % human prescriptions, antibiotics where cephalosporin was the highest followed by quinolones, penicillin, aminoglycosides and etc. Recent studies on human antibiotics revealed similar results in the context of Bangladesh [[44], [45], [46], [47]] Cephalosporin is a beta lactam antibiotic, active against a wide range of infections and it's generation variation may prefer it to be prescribed as the topmost antibiotic. Due to the Covid-19 pandemic, the pattern of antibiotic usage has increased and changed as penicillin or carbapenems were used massively in respiratory infections [48,49]. In the Indian subcontinent, excessive use of the antibiotic phenomenon has been observed in Pakistan and India with continental cultural similarities [[50], [51], [52], [53], [54]].

In Bangladesh, research data providing information on antibiotic consumption pattern in animal has been inadequate [25]. In our study, around 23.64 % of prescriptions got antibiotics where cephalosporin was the top in veterinary practice but sulpha drug and tetracycline were used only in animals. The veterinarian may also choose cephalosporins like the physicians but sulpha drug and tetracycline both prevent the synthesis progression incorporate with dihydropteroate synthase (DHPS) enzymatic activity and ribosomal translation respectively. Though a few research findings revealed the antibiotic prescribing rate but differ in antibiotic usage coherences [[55], [56], [57]]. Aminoglycosides were prescribed most but in risk assessment penicillin showed a strong association in relation to prescription and patients (Human and animals). Antibiotic use pattern may change in different areas based on animal, pathogen virulence, owners’ manner, farm managements, geo-climatic conditions and etc. Veterinary antibiotics has been used tremendously in livestock practices in India also [58,59]. We observed that 11.02 % veterinary prescriptions prescribed the β-lactam inhibitor for better result which indicates that extended spectrum of β-lactamase (ESBL) resistant pathogens have been persistent in Bangladesh [60,61]. So, it is high time to take one health approach to prepare for any upcoming antibiotic-resistant pandemics.

AMR is a multi-etiological phenomenon which initiates not only a complexity of pathological conditions but also challenging to set recovery strategies. The environment is considered as one of the important harboring sites of antimicrobial resistance pathogens due to antimicrobial residual issue, biocides, heavy metal pollution and etc. which initiates the AMR through co-resistance and cross-resistance phenomenon [4,62]. Migratory birds, cross-border wildlife movement and animal smuggling may also initiate AMR hazard to Bangladesh [[63], [64], [65]]. In this study, we got five resistant zones of inhibition for *E. coli* (AM, CIP, COT, CTR and GEN), two for both *Salmonella* sp. (AM and E) and *Staphylococcus* sp. (AM and CIP) respectively. In here, environment isolates were resistant to common antibiotics along with only human and veterinary antibiotics. Recent studies in Bangladesh on the same issue revealed similar findings of resistance phenomenon [[66], [67], [68], [69]]. Human may get exposure of extensive veterinary antibiotic resistant properties through vegetation and associated issues; vice versa may also occur in animals [70]^.^ On the other hand, the resistant pathogens are getting exposed frequently to human and animal in multiple transmission ways which is also a public health concern [71].

### 4.1 Limitations and recommendations

The samples analyzed in this study were restricted to the Mymensingh district, which poses a significant limitation due to the short duration of the study and the limited geographic scope. To fully comprehend antibiotic pollution's environmental impact and its correlation with prescribing patterns and multi-drug resistant microorganisms, it is necessary to expand research efforts beyond a single selected area within Bangladesh. Nevertheless, this study provides valuable insight into an integrated approach towards combating antibiotic resistance through analyzing both prescription practices and environmental factors. In future studies, additional environmental variables could be incorporated alongside diverse sampling locations and larger quantities of prescribing data to further enhance our understanding of this complex phenomenon. Awareness against the antibiotic using is a crying need for better health through incorporating the AMR issue in the textbook, campaigning programs in newsprint, online and offline platforms.

## 5 Conclusions

It can be inferred that AMR is poised to become the next pandemic, presenting a formidable challenge due to various socio-cultural and geo-climatic factors. Our study aimed to establish a correlation among the human-animal-environmental interface concerning antimicrobial resistance. At this critical juncture, it is imperative to take proactive measures towards reducing the overuse, abuse, and misuse of antimicrobials in human, animal, agriculture, and aquaculture practices. Adhering to the “one drug for one disease” principle could help prevent unnecessary exploration of AMR. This research has highlighted prescribing similarities and differences between human physicians and veterinarians while emphasizing their role in raising awareness about healthcare medicine choices in Bangladesh. Prescribers must adhere strictly to national guidelines on antimicrobial prescription while promoting hygiene practices that foster healthy ecosystems. Additionally, we sought to provide insight into how environmental factors drive AMR development - without a conducive environment for healthy living; achieving an infection-free lifestyle remains elusive. Our findings serve as a benchmark for understanding antibiotic stewardship trends both currently and in future times within Bangladesh's context.

## CRediT authorship contribution statement

**Zakaria Al Noman:** Methodology, Investigation, Formal analysis, Data curation, Conceptualization. **Tasnia Tabassum Anika:** Validation, Software, Resources, Methodology, Investigation. **Ummay Humaira Safa:** Methodology, Investigation, Formal analysis, Data curation. **Safaet Alam:** Resources, Methodology, Investigation, Data curation, Conceptualization. **Subarna Sandhani Dey:** Methodology, Investigation, Formal analysis, Data curation. **Md. Nurul Huda Bhuiyan:** Supervision, Software, Resources, Data curation. **Mahbubul Pratik Siddique:** Visualization, Validation, Supervision. **Md. Mahmudul Hasan sikder:** Visualization, Validation, Supervision.

## Declaration of competing interest

The authors declare that they have no known competing financial interests or personal relationships that could have appeared to influence the work reported in this paper.
